# Fission–fusion dynamics in sheep: the influence of resource distribution and temporal activity patterns

**DOI:** 10.1098/rsos.230402

**Published:** 2023-07-19

**Authors:** Katja Della Libera, Ariana Strandburg-Peshkin, Simon C. Griffith, Stephan T. Leu

**Affiliations:** ^1^ Department of Natural Sciences, Minerva University, San Francisco, CA, USA; ^2^ Department of Ecology and Evolution, University of Chicago Biological Sciences Division, Chicago, IL 60637-5416, USA; ^3^ Biology Department, University of Konstanz, Konstanz, Baden-Württemberg, Germany; ^4^ Centre for the Advanced Study of Collective Behaviour, University of Konstanz, Konstanz, Baden-Württemberg, Germany; ^5^ Department for the Ecology of Animal Societies, Max Planck Institute of Animal Behavior, Radolfzell, Baden-Württemberg Germany; ^6^ School of Natural Sciences, Macquarie University, Sydney, New South Wales 2109, Australia; ^7^ School of Biological, Earth and Environmental Sciences, University of New South Wales, Sydney, New South Wales, Australia; ^8^ School of Animal and Veterinary Sciences, The University of Adelaide, Adelaide, South Australia, Australia

**Keywords:** fission–fusion, movement, social behaviour, sheep, clustering, spatial dynamics

## Abstract

Fission–fusion events, i.e. changes to the size and composition of animal social groups, are a mechanism to adjust the social environment in response to short-term changes in the cost–benefit ratio of group living. Furthermore, the time and location of fission–fusion events provide insight into the underlying drivers of these dynamics. Here, we describe a method for identifying group membership over time and for extracting fission–fusion events from animal tracking data. We applied this method to high-resolution GPS data of free-ranging sheep (*Ovis aries*). Group size was highest during times when sheep typically rest (midday and at night), and when anti-predator benefits of grouping are high while costs of competition are low. Consistent with this, fission and fusion frequencies were highest during early morning and late evening, suggesting that social restructuring occurs during periods of high activity. However, fission and fusion events were not more frequent near food patches and water resources when adjusted for overall space use. This suggests a limited role of resource competition. Our results elucidate the dynamics of grouping in response to social and ecological drivers, and we provide a tool for investigating these dynamics in other species.

## 1. Introduction

Animal groups are common across taxa, yet vary in size, composition and temporal stability both within and across species. Understanding the drivers of such variation requires the consideration of the cost–benefit ratio of grouping [1]. Classic benefits of grouping include protection from predators and information transmission, while costs include increased intra-group competition for food and transmission of pathogens [2–5]. Social behaviour evolves when the fitness benefits of grouping outweigh the costs. Importantly, the costs and benefits vary as a function of group size [6–10]. From this it follows that an optimal, intermediate group size should exist which maximizes the net benefits [11,12].

In many species, group size and composition vary over time through large groups splitting and small groups merging. Such *fission–fusion dynamics* can occur across multiple time scales—from seasonal fluctuations to daily and even more frequent changes. Fission–fusion dynamics allow animals to flexibly adjust their group size and composition in response to currently experienced environmental conditions [11,12]. Studying fission–fusion dynamics and group size can thus give insight into the ecological drivers of social behaviour. We expect smaller groups and increased rate of group fission when the costs of grouping outweigh the benefits, for instance when within-group competition is high. Whereas, when benefits are high, such as during periods of greater predation risk, we expect larger groups. Fission–fusion behaviour has been linked to patchy resource distribution in monkeys [13], mitigation of infection risk in bats [14], building of social relationships in elephants [15], and responding to changes in food availability in ancient hunter–gatherer societies [16]. Fission–fusion dynamics are also common in herding ungulates such as sheep, horses or giraffes, many of which are of economic importance [17–19]. Furthermore, where and when fission–fusion events occur can also provide some insight on potential drivers. For example, fission–fusion events happening predominantly during grazing periods or at particular locations, such as clumped food resources, would indicate a larger role of resource competition as a driver. Conversely, fission–fusion events that occur during resting periods of the day when food competition is reduced, could indicate that predation risk, and safety in numbers, plays a role.

GPS tracking technology offers possibilities to investigate fission–fusion events through continuous movement tracking of all group members. However, this approach is based on defining a social group based on locational data of all group members over time. It further requires making some biologically meaningful assumptions about interindividual interactions, particularly at which spatial distance they occur [20] while accounting for characteristics of the used technology. When using GPS technology this includes the precision of each location as well as potentially missing data points. Once groups and their members are identified at each step in time, fission and fusion events can be deduced.

Here we fitted GPS collars to all members of sheep (*Ovis aries*) social groups in a paddock with known resource distribution to monitor their movements continuously over 16 consecutive days. Domestic sheep offer a uniquely tractable, and economically important, system for studying fission–fusion dynamics. They are highly gregarious and show frequent changes in group composition, allowing for the observation of many fission–fusion events on a practical time scale for experimentation. Understanding sheep flocking and fission–fusion behaviour also has potential benefits for their management, for instance establishing optimal group sizes in given paddock conditions.

Using the temporal and spatial information of the GPS tracking data, we identified group sizes and fission–fusion events in free-ranging sheep for each time step and mapped them across space and time. We hypothesized that group sizes and fission–fusion frequencies are variable in space and time, reflecting resource distribution and sheep activity patterns. This provides important insight into the factors that drive fission–fusion events in sheep and ultimately the dynamic cost–benefit trade-off that drives social grouping and group size.

## 2. Methods

### 2.1. Study site and animals

The study was conducted at Fowler's Gap Arid Zone Research Station in Australia, in 2018. Our study tracked a herd of 50 female merino sheep (*Ovis aries*) interacting in a paddock over 16 days. The paddock did not include any other conspecifics, and was approximately 6 km^2^ in size. It contained one water trough and 10 food locations consisting of bales of hay. The region was severely affected by drought and most of the annual vegetation that sheep would graze on was absent. Thus, the provided hay constituted the vast majority of food resources available to the study animals. The perennial vegetation in the area consisted primarily of blue bush (*Maireana* sp.) and small trees (*Acacia* sp.) [21]. The area can be subject to occasional sheep predation events by stray dogs. No predation occurred during our study and dingos are excluded by the dingo fence [22]. We recorded the locations of all 50 sheep every 6 s for 24 h each day using GPS collars. Every 4 days, sheep were captured to change GPS collar batteries. The data from these capture time periods were excluded from the analysis. Sheep were undisturbed during the 4-day tracking periods (86 to 88 h each). All sheep were born in 2016 and were approximately the same age. In the weeks before the study, they were kept together as a small herd and had the opportunity to interact with each other. One sheep died of natural causes at the end of the first 4-day period and was replaced by a different sheep for the remaining 12 days. GPS collars (GPS, i-gotU GT-120 by MobileAction, with a larger battery CE04381 by Core Electronics) weighed 700 g, approximately 1.9% of the sheep's body mass (mean = 36.5 kg, SE = 0.6 kg), falling well below the common 5% threshold for fitting scientific devices [23]. Hobbs-Chell *et al*. [24] showed that carrying data loggers does not affect sheep behaviour, and we therefore assume that this was also the case in our study group.

### 2.2. Processing the GPS data

We followed the data processing procedure described in Leu *et al*. [25] to increase overall data quality. Briefly, we first plotted all GPS locations and removed obvious outlier locations outside the paddock boundaries. We also removed all locations that were determined with fewer than three satellites, which is the minimum required for triangulation. Second, we removed locations that could not have been reached if the animal moved at maximum speed, taking the two previous and two following locations into consideration. We used a maximum movement speed of 1.5 m s^−1^, which was determined in a simulated predation event [26]. Lastly, because GPS units differed slightly in their recording times for each fix, we linearly interpolated the data such that each sheep's location was estimated at exactly 6 s intervals. We also filled small gaps in the data using the data interpolation (maximum of two consecutive missing locations). The processed data comprised 10 362 043 data points, representing 97% of the possible maximum number of data points across all sheep and time steps.

### 2.3. Defining groups from GPS data

We inferred group membership over time based on two steps: (i) spatial clustering of individuals into groups at each time step and (ii) connecting groups across time steps by defining what we considered to be the same group. We determined groups at each time step, using a modified version of the density-based spatial clustering of applications with noise (DBSCAN) algorithm [27].

### 2.4. Defining groups at each time step

Groups are built through an iterative process, based on DBSCAN. We outline the original DBSCAN before adding our modification. The algorithm starts with a randomly picked focal sheep and connects all sheep within the given radius to the focal sheep and to each other. This approach reflects the ‘gambit of the group’, an assumption that all individuals in a group are associated with each other, thus ignoring internal group structure [28]. Then, the algorithm identifies and connects all further individuals that are within the radius of any group member. This is repeated until no additional sheep can be added to the group. Then, a new focal sheep, not yet a member of any group, is chosen, and the iteration starts again. The process is repeated until all sheep are assigned to a group or are identified as unconnected solitary individuals. The algorithm returns a list of the groups for each time step. In its original form, the inputs to DBSCAN are (i) a radius that describes the maximum Euclidean distance between two individuals considered associated with each other, and (ii) an integer representing the minimum group size. In our study, we set the minimum group size to 1, thus considering groups of any size.

Different radii (interindividual distance thresholds) capture different interaction types and may result in different group compositions. For instance, physical contact occurs at shorter distances than social interactions through visual or auditory communication. One or two body lengths are often used to determine social interactions in animals [29–31]. In sheep, 3 and 5 m have been used [30,32] to capture social interactions at close proximity. However, the auditory and olfactory senses of sheep detect conspecifics significantly farther apart than 5 m [20]. Hence, in other contexts, such as grazing, the sheep are physically farther apart but are still able to perceive cues from other group members, for instance after detecting a predator, and display some cohesion by moving together. Even so, group members rarely move in the average direction of the group but rather with large variation along a common route [33] making the 3–5 m radius inappropriate to capture these dynamics. We were interested in these larger groups providing benefits for the individual, rather than physical interactions among group members and thus used an inner radius of 30 m. In addition, we conducted a robustness check using radii between 5 and 60 m displayed in electronic supplementary material, figures S1–S6.

### 2.5. Double-radius (sticky) DBSCAN

**Figure 1.**
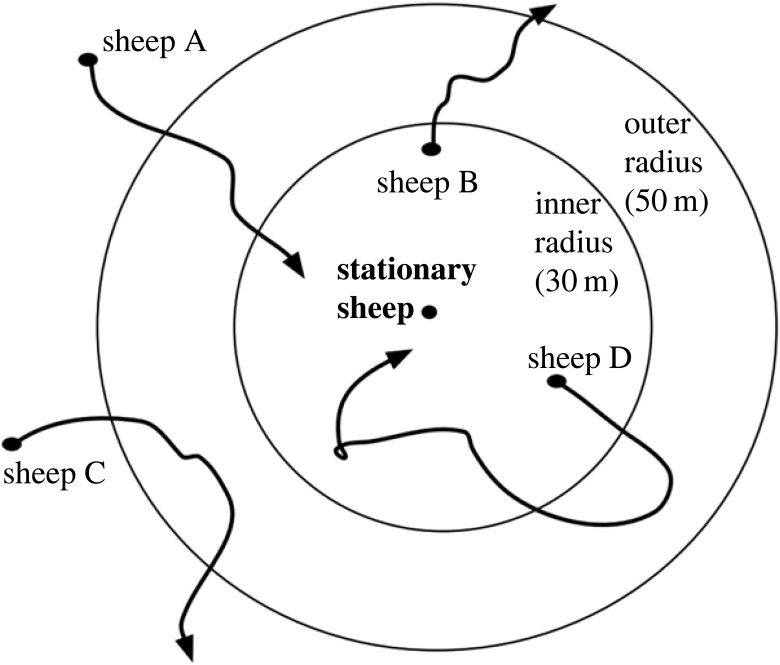
The ‘sticky DBSCAN’ algorithm uses the current location of the sheep relative to another sheep to determine group membership. In this example, sheep A joins the stationary sheep when it passes the outer radius because it also passes the inner radius. Sheep B leaves the stationary sheep when it reaches the outer radius, and sheep C never joins the stationary sheep because it does not pass the inner radius. Sheep D passes the inner radius but not the outer radius so it is considered to remain grouped with the stationary sheep. In this example we only consider the stationary sheep and sheep A through D for illustrative purposes; however, the algorithm repeats the analysis for all combinations of sheep. Furthermore, though the example here uses a stationary individual for simplicity, the algorithm operates based on distances between individuals and therefore will also detect groups when all animals are moving.

Defining membership in a social group based on a spatial radius can result in group membership fluctuating with small changes in the distance between individuals, although the social link is probably still present. Such fluctuations can be problematic in the context of identifying fission–fusion events, because they can lead to the detection of many small events that are not biologically/socially meaningful. To limit the effect of such fluctuations, we applied a ‘sticky DBSCAN’ algorithm, which uses two radii—an *inner* radius and an *outer* radius. An individual is considered to join another individual in a group only if their dyadic distance is smaller than the inner radius. However, the time at which the individual joins is identified as the moment when the dyadic distance falls below the outer radius (figure 1). Similarly, an individual is considered to leave the group at the time it crosses the outer radius of all group members after it has previously been within the inner radius. A dyadic version of this algorithm has been used to identify fission–fusion events in hyenas [34]. Here, we extend it to a group context. We assign group membership to all tracked animals, and determine fission–fusion events between groups of variable sizes, rather than focusing on fission–fusion events between pairs of individuals, only.

The sticky (double-radius) DBSCAN algorithm increases the detected group stability as illustrated in figure 1. Compared with the traditional (single-radius) DBSCAN with radius 50 m, stability arises from sheep C never being considered joining the group, and for radius 30 m, sheep D never being considered leaving the group.

### 2.6. Identifying fission–fusion events

**Figure 2.**
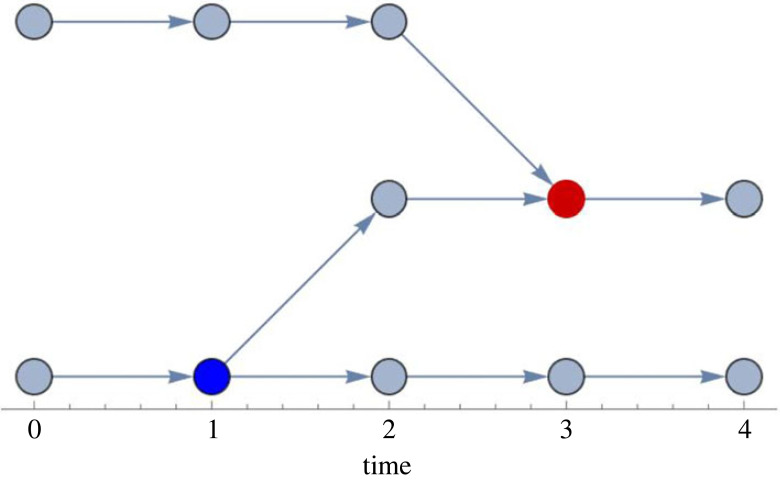
An example network arranged on a timeline showing a fission (blue) and a fusion (red) event. Each node represents a sub group at a given time point. We use the larger group as a reference point to identify the time step of a fission or fusion event. The time step in which the larger group still existed determines the fission time, and the time step the larger group formed determines the time of the fusion event. Here the vertical arrangement is optimized for visibility.

After identifying groups at each time step, we represent them as nodes in a directed graph. Edges in this graph represent overlap in the members of groups in consecutive time steps. Arranging the groups on a timeline as seen in figure 2 allows us to identify where unique groups start and end through fusion (red) and fission (blue) events. When two or more edges converge on a single node in the next time step, this indicates a fusion event. Conversely when a single node spawns two or more edges this represents a fission event. Nodes connected by a single edge represent a group that has remained stable.

We define a fission and fusion event if one or more individuals left or joined the group, respectively. An individual can temporarily leave one group and return to the same group without having joined another group in between. The algorithm recognizes this as a fission event when the individual leaves the group, a new ‘group’ of size 1, and finally a fusion event when the individual re-joins. In the rare cases of missing data, missing individuals are not considered as having changed group membership, and individual ‘disappearances’ and ‘reappearances’ in the dataset are not considered as fission–fusion events.

### 2.7. Spatial and temporal patterns of fission–fusion events

We determined the spatial location of the fission or fusion event as the mean of the locations of all sheep present in the group.

In addition to the events themselves, to investigate temporal patterns in group size, we computed the average group size a sheep experienced using the equation

$$\frac{\left(\sum_{i=1}^{n}N_{i}*N_{i}\right)}{\left(\sum_{i=1}^{n}N_{i}\right)},$$

where *N_i_* is the size of group *i*, and *n* is the number of groups. The term $\sum_{i=1}^{n}N_{i}$ is the total number of sheep across all groups. This metric was chosen because it represents the average group size as experienced by an individual in the population, as opposed to the average group size as seen from the perspective of an observer. We chose to represent the typical group size experienced by individuals because this perspective is more biologically relevant, as it captures the information that individuals in a population might consider when making behavioural decisions. For example, if a population of 4 individuals consisted of a group of size 3 and a group of size 1, the average experienced group size would be computed as (3 × 3 + 1 × 1) / 4 = 2.5, because 3 sheep experienced a group size of 3 (3 × 3) and 1 sheep experienced a group size of 1 (1 × 1). We then assessed temporal patterns in average experienced group size as a function of time of day by calculating the mean across the 16 days for each minute of the day. Next, we split the data into night (22.00–5.00), morning (5.00–11.00), midday (11.00–16.00) and evening (16.00–22.00). This split is an approximation based on dawn (approx. 5.00) and dusk (approx. 21.00), which also captures different activity levels of sheep [25]. We looked at the distribution of average experienced group size in each time of day bin.

We investigated whether fission–fusion events were more likely at certain times of the day. Furthermore, to explicitly relate fission–fusion frequencies to physical activity and motion, we calculated the Euclidean distance every sheep moved during each 6 s measurement interval. Then for each minute of the day, we calculated the mean motion per 6 s (m) by averaging across all measures per minute, and across all sheep and 16 days, resulting in a metric that captures population-level activity for each time point. We then normalized the fission–fusion events by activity level. To this end, we divided the number of fission–fusion events by the average motion for the same minute. Investigating the temporal pattern of the normalized measure allowed us to determine whether fission–fusion events are a linear function of activity level alone or still vary over time.

We also investigated whether fission–fusion events were more likely to occur close to known food and water resources, to assess the role of resource competition as a driver of fission–fusion event dynamics. First, we superimposed a 50 × 50 m grid over the paddock. We chose this grid cell size to be consistent with the radius used in the modified DBSCAN algorithm. For each grid cell, we determined the number of fission or fusion events that occurred within it during the 16-day study period. We then calculated the distance from the cell's central point to the nearest food or water source. As a measure of propensity for fissions and fusions to occur in a given location, we counted the total number of fission and fusion events that occurred in each 50 × 50 m grid cell and divided this number by the number of time steps sheep spent within that cell. This normalized value represents the frequency of fission or fusion events relative to the overall use of that particular cell. We only considered grid cells that sheep used during at least 50 time steps to account for higher uncertainty at lower sample sizes.

Finally, we investigated the relative importance of space and time in driving fission–fusion dynamics by fitting a regression model to the number of fission–fusion events for each 50 × 50 m grid cell for each hour of the day. We used the lmer function in the lme4 package in R for our analysis [35]. To determine the importance of space we included the distance to the closest food or water source as one explanatory variable in our model. As a proxy for the temporal pattern of sheep behaviour we included the mean activity level per hour as the second explanatory variable. Sheep movement activity varies over time, with periods of higher activity in the morning and evening, and with ambient conditions [25]. Because fission–fusion events can vary with activity level [36], and because activity level changes non-monotonically throughout the day (figure 6), we included activity level directly as an explanatory variable instead of hour of the day. Here, we calculated the population level activity for each hour of the day as the mean distance moved by each sheep per 6 s time step. We also included the interaction term of distance and activity in our model. All independent variables were scaled using the transform and scale function in R. We added the grid cell ID as a random intercept to account for spatial characteristics that are not captured by distance to resources. Results were extracted using the Anova function in the car package which uses the Wald test [37]. The model took the form

fission−fusion events∼distance+activity+distance×activity+(1|grid cell ID).

We then ran the same model using the fission–fusion rate normalized by space use as the dependent variable. Therefore, we divided the number of fission–fusion events per hour in each grid cell by the overall use of the grid cell, i.e. across the 16 days. The rest of the model remained the same,

normalized fission−fusion events∼distance+activity+distance×activity+(1|grid cell ID).

## 3. Results

**Figure 3.**
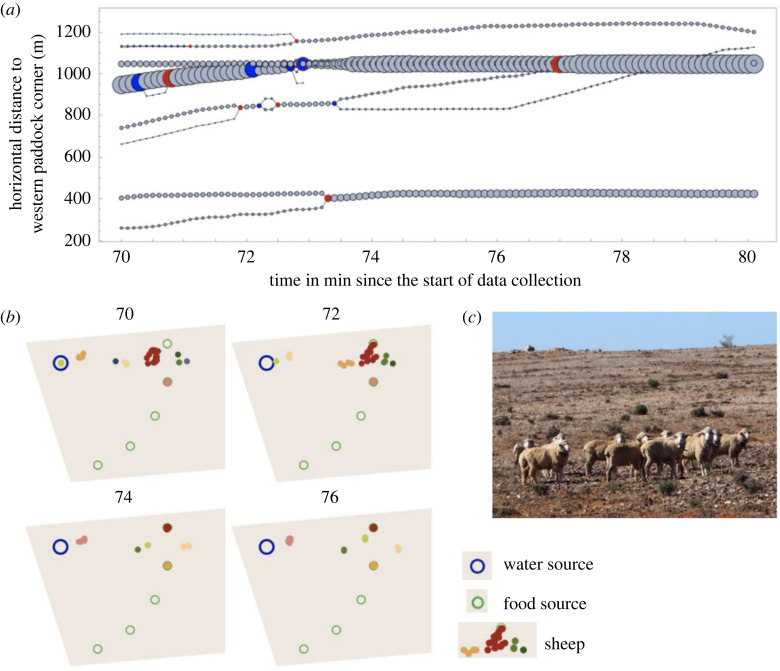
A plot showing the network output of the algorithm for an example period of 10 min, 70 min after the initial start of the measurements. Groups start in different locations and merge or dissolve over time. Nodes represent subgroups. Node size indicates subgroup size and coloured nodes are classified as fusion (red) or fission (blue) events. We calculated the value on the *y*-axis as the distance between the central point of the group and the westernmost paddock corner. This measure only serves to spatially separate the points in order to illustrate fission–fusion events across time, but has no further meaning. (*b*) Maps showing the actual sheep movement during the 10 min displayed in (*a*). Sheep further west on the map in (*b*) are lower on the y-axis of the plot in (*a*). Colours distinguish different groups. (*c*) Sheep wearing GPS collars at Fowler's Gap.

The clustering and group continuation algorithm together created a network whose nodes represent a specific group at a specific time step (example in figure 3).

Using the sticky DBSCAN algorithm with an inner radius of 30 m and an outer radius of 50 m we identified a total of 6831 fission–fusion events across all 50 sheep and over the course of 16 days. By contrast, the original DBSCAN with a single radius of 50 m identified 18 783 fission–fusion events, and 46 002 events with radius 30 m. As expected, the single radius method identified many more fission and fusion events.

### 3.1. Temporal pattern of fission and fusion events

**Figure 4.**
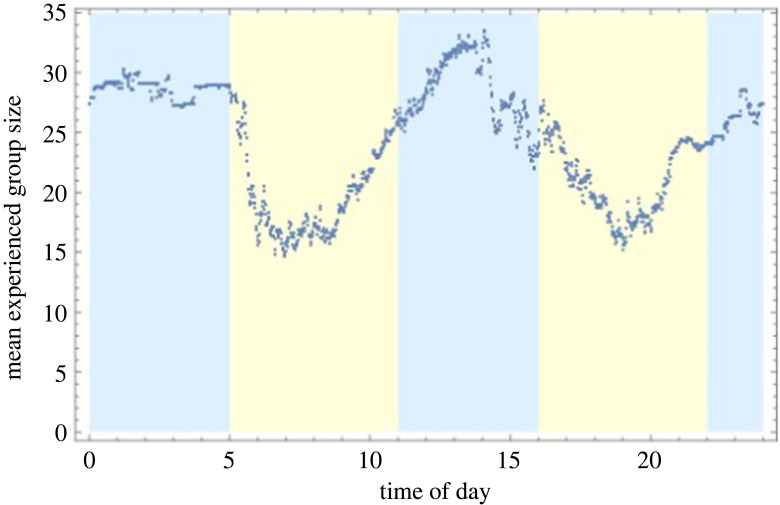
Mean experienced group size across all 16 days of data, as a function of time of day shows clear variation.

We found that sheep experienced larger group sizes during the night and midday period when averaging across days (figure 4).

**Figure 5.**
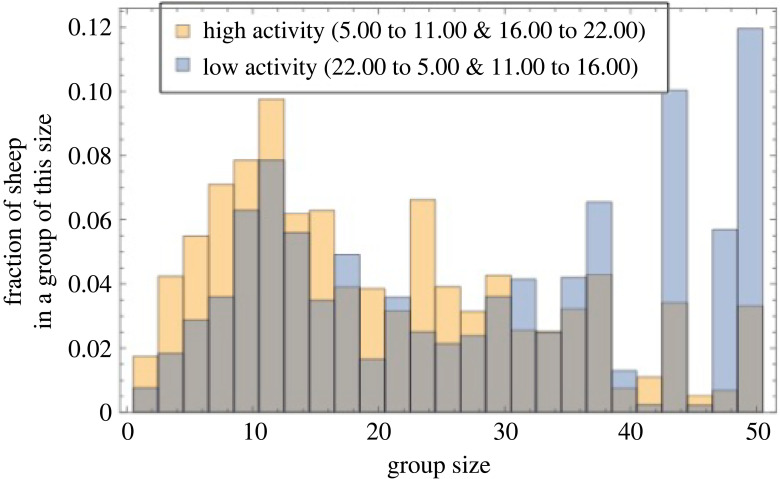
Relative frequency (proportion of observations) of experienced group sizes during periods of high activity (yellow) and periods of low activity (blue), which reflect rest at night and rumination around mid day.

During periods of high activity (5.00–11.00 and 16.00–22.00, shaded yellow) the most commonly experienced group size was 12 (mode) and the mean was 20.43 (95% confidence interval: 20.42 to 20.44). However, during periods of low activity (22.00–5.00 and 11.00–16.00, shaded blue), the group size distribution was bimodal with peaks at 12 and 50 (i.e. all) individuals (figure 5). The mean experienced group size was 28.09 (95% confidence interval: 28.07–28.10).

**Figure 6.**
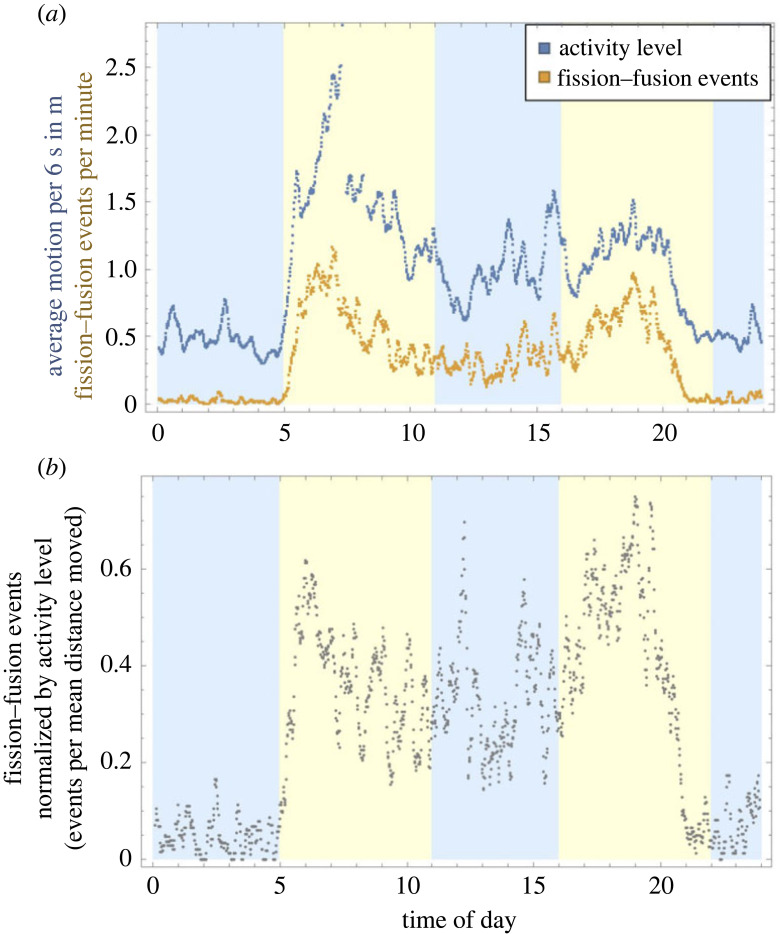
(*a*) Comparison of overall sheep activity level and number of fission–fusion events. Orange points indicate the mean number of fission–fusion events occurring in the group per minute and blue points indicate the mean distance covered by individual sheep in a 6 s period, with means calculated for each minute across all sheep and all 16 days of data collection. Curves are smoothed by taking a moving average with window size 10. (*b*) The same data of fission–fusion events divided by the activity level. Activity level was calculated as in (*a*). The curve is smoothed by taking a moving average with window size 10.

Overall, fission and fusion events occurred more frequently early and late during the day. The frequency was lowest at night, with another low around midday (figure 6*a*). The pattern of fission–fusion events over time closely follows the average activity level, measured as average distance of motion (figure 6*a*). However, some spikes in activity did not immediately lead to fission–fusion events, such as during the night and early morning a pattern that becomes more clear when looking at the rate of fission–fusion events per average motion (figure 6*b*). Activity levels increased during most of the midday period (11.00–16.00), but fission–fusion events did not show the same increase. In addition, while there is an increased level of fission–fusion events during the afternoon compared with the noon rumination period, the level of movement is similar.

### 3.2. Spatial pattern of fission–fusion events

**Figure 7.**
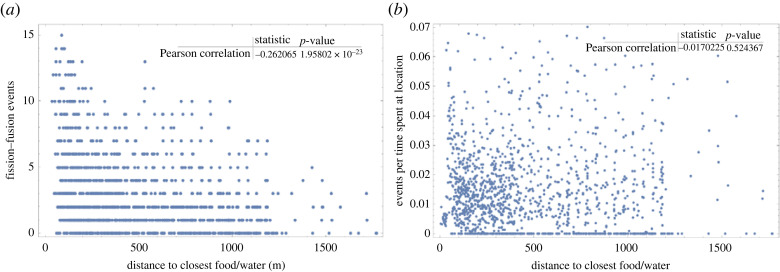
Plots showing the spatial distribution of fission–fusion events is an effect of space use. (*a*) Number of fission and fusion events negatively correlates with the distance to the nearest food or water source (Pearson's *R* = −0.26, *p* < 0.001). Each data point represents a fission–fusion event that took place in a single grid cell, as shown in electronic supplementary material, figure s1. (*b*) The normalized number of fission–fusion events does not significantly correlate with the distance to the nearest food or water source (Pearson's *R* = −0.02, *p* = 0.52; same data as in (*a*), but normalized by the time sheep spent in each cell).

Fission and fusion events occurred more frequently closer to a food or water source (figure 7*a* and electronic supplementary material, figure S1b). However, these locations were also used more frequently. When we normalized the data by the frequency of use for a given grid cell, the rate of fission or fusion was not influenced by the distance to food or water sources (figure 7*b* and electronic supplementary material, figure S1c).

The regression model shows that an increase in activity level and a decrease in distance to the closest food or water source both led to a statistically significant increase in the number of fission–fusion events (*p* < 0.001, table 1). Since both variables were scaled the estimated coefficients show that the effect of the distance was much stronger. The interaction term between the two variables was also significant (*p* < 0.001). When normalizing by the use of each square, distance to resources is only significant as an interaction term with activity.

**Table 1.** The results of the regression model including population activity level per hour, distance to closest food or water source, their interaction term and a random effect for the paddock grid cell. The left side of the table shows results from modelling the number of fission–fusion events and the right side shows results from modelling the normalized fission–fusion rate (i.e. scaled by overall space usage).

variable	number of fission–fusion events linear model			normalized number of fission–fusion events linear model		
random effects						
	estimate	variance	standard deviation	estimate	variance	standard deviation
grid cell ID (intercept)	0.0000	0.1965	0.4433	0.0000	0.003601	0.06001
residual	0.7849	0.7849	0.8859	0.9959	0.995853	0.99792
fixed effects						
	estimate	Chi squared	*p*-value	estimate	Chi squared	*p*-value
activity	0.0315	42.509	<0.001***	0.0207	14.4870	0.0001***
distance	−0.1299	103.166	<0.001***	−0.0036	0.406	0.5243
activity × distance	−0.0329	46.420	<0.001***	−0.0140	6.655	0.0099**

## 4. Discussion

Sheep experienced larger group sizes during the night and midday periods, which are both times of rest [25]. During the midday period sheep often ruminate [38] or spend time at water points [25], and at night they rest [39]. During these non-foraging periods the net benefit of grouping is higher because food competition within the group is limited or absent, reducing grouping costs, while anti-predator benefits such as shared vigilance and the dilution effect remain [40,41]. Sheep predation events are rare on the dingo exclusion side of the dingo fence but even occasional predation events by stray dogs and evolutionarily conserved predator avoidance can affect behaviour [22,42,43]. Mouflon (*Ovis orientalis*), the wild ancestors of all domestic sheep (*Ovis aries*), experienced predation for example by wolves in their natural mountain habitat in the Lesser Caucasus and Zagros Mountains [44,45].

During periods of activity, the most frequent behaviour is grazing, searching for food and moving to known resource locations [46]. Hence, when food availability is limited, such as during drought conditions, active periods are associated with increased resource competition [47]. This can explain the lower experienced group sizes during periods of activity and the increase of fission–fusion events when the sheep are active. When normalized by activity levels, the rate of fission–fusion was still high at the start and end of the day with some additional higher rates at the start and end of the inactive period in the middle of the day. This could suggest active decision-making during those times, rather than an effect of activity levels alone. Nonetheless, our model showed that the number of fission–fusion events increased with the mean activity level of the study population. Maintaining group cohesion during activity and when moving is more difficult and ultimately more costly than when groups are inactive and stationary, which could result in higher fission–fusion frequencies when active. Our results are consistent with studies of smaller groups of sheep which showed that groups are most cohesive during periods of rest and that cohesion and thus group size decreased when some of the sheep were active [36]. This pattern is found in other mammals as well, for example baboons, which sleep in larger colonies and forage in smaller groups [48]. Interestingly, the opposite has been found in some species of fish, which also show collective movement. Here, many species display schooling during the daytime but not at night, potentially because of the inability to coordinate movement without light [49,50]. By contrast, sheep rarely move at night [51], but aggregating in larger groups may provide protection from nocturnal predators when visibility for the sheep is reduced.

While sheep fission–fusion frequencies showed temporal patterns that can be explained by active and inactive periods, their rate of occurrence was more evenly distributed across space when adjusted for overall use. Food or water locations were local hubs of fission–fusion events in terms of raw frequency, but when accounting for the higher use of these areas, fission–fusion events were not more common than would be expected. During our robustness check with varying radii, we found that this pattern of higher overall numbers of fission–fusion events near resources was consistent across different radii used to define group membership (electronic supplementary material, figures S3–S6). The linear model shown in table 1 also showed the effect of distance in reducing fission–fusion events, as well as the positive effect of increased activity. The estimated coefficients for the scaled activity level and scaled distance show that the effect of the distance was stronger. The interaction term between the two variables was also significant (*p* < 0.001). That is, near resources and at high activity level fission–fusion events were more common, but at larger distances from the food or water source the activity level had a reduced effect as generally fewer fission–fusion events occurred. When normalizing by the use of each square, distance to resources is only significant as an interaction term with activity, meaning that at larger distances to resources the effect of activity is still reduced, but distance alone had no significant effect. Taken together, our results suggest that resources (food or water) are important spatial drivers of the number of fission and fusion events because they result in high use of these areas, but competition for those resources may not be an important social driver of the fission–fusion rate when corrected for this space use. Whether this result can be generalized to other food patch distributions or other species remains to be investigated and is an interesting future direction for the study of fission–fusion behaviour. The overall tendency to merge or split at certain times of the day, which coincides with shifts between activity and inactivity, suggests that shared vigilance and safety in numbers, and hence anti-predatory benefits, are more important drivers of sheep fission–fusion behaviour and group size than resource competition. Actual levels of predation are low in our study area, hence we suggest that these behaviours mainly reflect evolutionarily conserved predator avoidance [43]. Nevertheless, other unmeasured features of the environment, such as vegetation that provides shade could still influence the rate of fission–fusion events. For instance, Strauss *et al*. [34] showed, using a similar double radius method, that in spotted hyena (*Crocuta crocuta*) fission–fusion events occur preferentially at communal den locations, where cubs are raised. All group members visit these sites for social interactions even if they are not nursing cubs [34]. In hamadryas baboons, there are specific sleeping sites on the top of cliffs from where groups fission in the morning and congregate at night [48]. Although our study did not identify locations such as the dens in hyenas or sleeping sites in baboons, we showed that fission–fusion events were most common at the start and end of the daily activity (figure 6) which would be at or close to the locations of overnight rest. This suggests that times when activity levels change such as in the morning and at night, and the associated locations, are times and places of social reorganization. This could potentially be further modulated by differential hunger levels among individuals after rest at night.

Our study showed the importance of fission–fusion dynamics in adjusting group size to different needs over the course of the day, driven by resource needs and predator vigilance.

Methodologically, our study contributes a simple and general approach to identifying subgroups and fission–fusion events from continuous movement data of animal groups. Our approach can be adapted to suit different questions about drivers of fission–fusion dynamics on different spatial and temporal scales. For example, by choosing a smaller radius it would be possible to investigate the biological drivers of closer social interactions involving physical contact, while choosing a larger radius would enable investigation of social interactions over larger distances, such as vocal communication in dense habitats that restrict visual contact. Furthermore, taking a similar approach across a longer observation period would allow investigation of additional questions, for instance seasonal patterns, shedding light on how environmental changes shape the costs and benefits of grouping.

Compared with the original algorithm, the double radius (sticky) version of DBSCAN presented here identifies fewer fission–fusion events resulting in a group stabilizing effect. This represents a conservative approach as it only identifies fission–fusion events reflective of greater attraction to and greater repulsion from the group because two radii have to be crossed. Focusing on events of greater attraction and repulsion allows us to determine the fundamental drivers of fission–fusion events more robustly. Nevertheless, the strength of social attraction and repulsion is probably heterogeneous among individual sheep. The algorithm we developed here not only captures fission–fusion events in space and time, but could also be used to collect information about the individual members of each group, such as individual-level movement relative to group characteristics, which individuals are more likely to join or leave subgroups, or whether certain subgroups repeatedly reoccur over time. These future directions would provide important additional information on fission–fusion behaviour and its relationship to individual-level decision-making and social relationships, complementing our population level perspective in the current study.

Future improvements of the algorithm could also consider additional criteria in determining an association between two individuals, such as their orientation to each other and speed of motion at each time step. A potential alternative to the spatially sticky DBSCAN is a temporally sticky version. For instance, an individual could be required to remain outside a single radius for a specific period of time before being considered as having left the group. For example, Sankey *et al*. [52] discarded fission–fusion events where an individual pigeon left the flock for less than 2 s.

Ultimately, spatial proximity over time is only a proxy for social interactions, and the types of social interactions (affiliative, agonistic) captured by proximity-based methods remain unidentified. With the further development of other observation methods (e.g. video drones, accelerometers) these more detailed components of the social interaction could be integrated into the definition or characterization of grouping patterns, providing further insight into social group formation and fission–fusion dynamics.

## 5. Conclusion

In this study we used a double radius DBSCAN in a group context to identify (i) the experienced group sizes and (ii) fission–fusion events of sheep, as well as where and when these events occurred. The spatial and temporal investigation of the group size and fission–fusion events allowed us to deduce potential drivers of those events. Experienced group size was largest during periods of inactivity, whereas fission and fusion frequencies were higher during periods when sheep were active. In particular, fission and fusion frequencies were highest early in the day and late in the afternoon, even when scaled for activity. This finding suggests that the start and end of daily activity is a period of social reorganization. Furthermore, fissions and fusions were not more frequent than expected near food or water resources when accounting for the higher frequency of use of those locations. Hence, intra-group competition is unlikely to be a driver of individual fission or fusion events at those locations. Overall, our study provides an example of context-dependent changes in group size, leading to fission–fusion events at certain times of the day. Mapping fission–fusion events in time and space provides deep insights into the social dynamics of animal groups and importantly may indicate potential drivers of social change.

## Data Availability

Data have been uploaded to Dryad Digital Repository: https://doi.org/10.5061/dryad.59zw3r2d6 [53]. Code: code can be found in the electronic supplementary material [54]. Software: R: https://www.r-project.org. Wolfram Mathematica: https://www.wolfram.com/mathematica/. Publicly available Sticky DBSCAN implementation (retrieve in notebook as ResourceObject [‘KatjaDellaLibera/StickyDBSCAN’]) or directly at https://resources.wolframcloud.com/PacletRepository/resources/KatjaDellaLibera/StickyDBSCAN/.
