# Correction to: ‘Fission–fusion dynamics in sheep: the influence of resource distribution and temporal activity patterns’ (2023) by Della Libera *et al.*

**DOI:** 10.1098/rsos.231083

**Published:** 2023-08-14

**Authors:** Katja Della Libera, Ariana Strandburg-Peshkin, Simon C. Griffith, Stephan T. Leu

*R. Soc. Open Sci.* **10**, 230402. (Published online 19 July 2023) (https://doi.org/10.1098/rsos.230402)

Owing to an error in the code, the activity-level data used in the linear-mixed model was incorrect changing the quantitative results of the model. All other activity measures used throughout the publication were unaffected and remain correct. Here we correct the values in table 1.

In the Discussion of the original paper, we compared the effect size of distance and activity: ‘The estimated coefficients for the scaled activity level and scaled distance show that the effect of the distance was stronger’. With the corrected values, the impact of these factors is comparable.

**Table 1.** The results of the regression model including population activity level per hour, distance to closest food or water source, their interaction term and a random effect for the paddock grid cell. The left side of the table shows results from modeling the number of fission–fusion events and the right side shows results from modeling the normalized fission–fusion rate (i.e. scaled by overall space usage).

variable	number of fission–fusion events linear model			normalized number of fission–fusion events linear model		
**random effects**						
	**estimate**	**variance**	**standard deviation**	**estimate**	**variance**	**standard deviation**
grid cell ID (intercept)	0.0000	0.1974	0.4443	0.0000	0.0049	0.0703
residual		0.7626	0.8733		0.9638	0.9817
**fixed effects**						
	**estimate**	***χ* ^2^**	***p*-value**	**estimate**	***χ* ^2^**	***p*-value**
activity	0.1414	881.95	<0.0001***	0.1727	1040.25	<0.0001***
distance	−0.1299	103.17	<0.0001***	−0.0036	0.41	0.5243
activity × distance	−0.0586	151.30	<0.0001***	0.0395	54.30	<0.0001***

